# Amide proton transfer imaging-arterial spin labeling mismatch: a new imaging biomarker for pilocytic astrocytoma

**DOI:** 10.1038/s41598-023-43235-2

**Published:** 2023-09-29

**Authors:** Adhithyan Rajendran, Chidambaranathan Natesan, Prashanth Jawahar, Sushama Patil, Srinivas Chilukuri, Siddhartha Ghosh, Roopesh Kumar, Rakesh Jalali

**Affiliations:** Apollo Proton Cancer Centre, Chennai, India

**Keywords:** Biomarkers, Neurology, Oncology

## Abstract

We describe the potential utility of Amide Proton Transfer weighted (APTw) Magnetic Resonance Imaging and arterial spin labeling (ASL) in characterizing pilocytic astrocytoma (PA), a type of brain tumor that can be challenging to accurately diagnose and treat. The study included 50 patients with solid or predominantly solid intra-cranial and intra-axial tumors, with 25 patients diagnosed with PA and 25 patients diagnosed with other types of tumors. The study found that the APTw imaging-arterial spin labeling (ASL) mismatch is a new imaging biomarker that could be used to differentiate PA from other types of tumors with a high degree of sensitivity and specificity. The results suggest that APTw imaging and ASL may be useful in characterizing PA, potentially improving diagnosis and treatment planning for this type of brain tumor.

## Introduction

Pilocytic astrocytoma (PA) is a type of brain tumor that is classified as a grade I tumor by the World Health Organization (WHO)^1^. Although PA is generally considered a low-grade tumor, it can present with imaging features that mimic those of higher-grade tumors, making accurate diagnosis and treatment planning challenging^2^. This is particularly true in cases where PA demonstrates infiltration of surrounding tissues, intratumoral hemorrhage, intense enhancement on post-contrast images, or leptomeningeal dissemination^3^.

In recent years, researchers have explored the use of non-invasive imaging techniques to better characterize PA. One such technique is Amide Proton Transfer weighted (APTw) MRI, which is a contrast-agent free brain MR imaging technique that provides information about the cellular proliferation rate and tissue pH. APTw imaging is based on the measurement of the amide proton transfer (APT) effect, which reflects the presence of endogenous cellular proteins.

Recent studies have demonstrated the potential utility of APTw MRI in characterizing brain tumors. For example, a study published in the Journal of Neuro-Oncology in 2020 evaluated the utility of APTw imaging in differentiating between low-grade and high-grade gliomas. The study found that APTw imaging was able to accurately differentiate between the two tumor grades with a high degree of sensitivity and specificity^4^.

Another imaging technique that has been used to characterize brain tumors is arterial spin labeling (ASL), which is a non-contrast MRI perfusion technique that measures cerebral blood flow. ASL has been used to evaluate the degree of angiogenesis in brain tumors and has been shown to help in the grading of gliomas^5^.

To date, no studies have investigated the use of APTw imaging or ASL in characterizing PA specifically. However, given the promising results of these techniques in characterizing other brain tumors, it is reasonable to hypothesize that they may also be useful in characterizing PA.

## Materials and methods

### Subjects

The study was conducted with the formal approval of the institutional ethical committee for Biomedical research at Apollo Hospitals, Chennai. All activities adhered to applicable local and international guidelines for conducting clinical research. Written general informed consent was obtained from all participating patients. This retrospective study analyzed data from consecutive patient studies conducted between January 2019 and April 2023 at a single, reputable tertiary institution. By conducting the research at one institution, the imaging protocols and analysis methods were consistent throughout.

The study focused on patients who underwent both Amide transfer imaging (APT) and arterial spin labeling imaging (ASL). Specifically, it included patients with purely solid or predominantly solid (> 75%) intra-cranial and intra-axial tumors. To ensure the accuracy and reliability of the results, cystic lesions with a mural nodule and cystic lesions irrespective of the lesion types, were excluded, as they could have raised APT values and potentially confound the findings. The researchers meticulously selected these inclusion and exclusion criteria.

The final study group comprised 50 patients, with 25 diagnosed with histopathologically proven grade 1 pilocytic astrocytoma, confirmed through conventional histomorphology and molecular testing, including BRAF mutation analysis when feasible. The other 25 patients were diagnosed with various brain tumor types, including grade 2 and grade 3 glioma, ependymoma, medulloblastoma, diffuse midline glioma H3K27 altered, embryonal tumor with multilayered rosettes, pleomorphic xanthoastrocytoma, and atypical teratoid/rhabdoid tumors.

**Figure 1:**
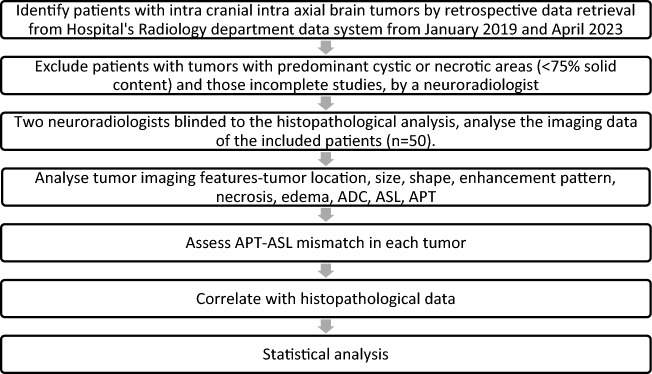
Flowchart: Study design.

Histopathological diagnosis and imaging data were retrieved from the hospital's electronic health record system, ensuring the accuracy and quality of the information used for the study. The imaging data was used to calculate the solid and cystic areas at the axial section of the maximum cystic region of the tumor, and the ratio of solid-cystic areas was determined by a radiologist for all brain tumors during the study period. Flow chart (Fig. 1), shows the clear representation of the inclusion and exclusion criteria and how the final study group was identified.

### MR imaging

The imaging was performed using a 3T MRI scanner, specifically the Ingenia Elition model made by Philips Healthcare, based in the Netherlands. This scanner utilizes a 32-channel receive-only head coil array for improved signal detection. The following sequences were acquired during the imaging process:

1. 3D Pseudo-continuous Arterial Spin Labeling (ASL) Perfusion Imaging: ASL is a non-invasive imaging technique used to measure cerebral blood flow in the brain. It employs magnetically labeled arterial blood water as a natural tracer to assess blood perfusion. The ASL was performed using a pseudocontinuous labeling method combined with a stack of spiral 3D fast spin-echo sequences. The imaging parameters used were as follows: Repetition Time (TR) = 4326 ms, Echo Time (TE) = 12 ms, Matrix = 64 × 60, Slice Thickness = 8 mm, Field of View (FOV) = 240 × 240 mm^2^, Total Scan Time = 5 min and 3 s, Post-labeling Delay = 2000 ms.
2. Amide Proton Transfer Imaging (APTw): APT imaging is a method used to provide information about cellular metabolism and tissue pH in the brain. It uses the two-dimensional single-shot fast spin-echo planner imaging (EPI) technique with a saturation pulse of 2 s and a saturation power level of B1, rms = 2.0 μT. The imaging is performed in a transverse oblique orientation parallel to the intercommissural line, acquiring 12 slices while excluding the paranasal sinuses to avoid artifacts.The imaging parameters used were as follows: TR = 5864 ms, TE = 8.3 ms, Matrix = 128 × 128, Slice Thickness = 6 mm, Field of View (FOV) = 230 × 180 mm^2^, Total Scan Time = 3 min and 37 s.
3. Diffusion-Weighted Imaging (DWI): DWI is a technique used to visualize the movement of water molecules in brain tissues, providing insights into tissue microstructure. The imaging is performed in the axial plane using a single-shot spin-echo echo planar imaging sequence. Two b-values are used: b = 0 s/mm^2^ (no diffusion weighting) and b = 1000 s/mm^2^. The apparent diffusion coefficient (ADC) is calculated through monoexponential fitting with the pair of b-values. The imaging parameters used were as follows: TR = 5826 ms, TE = 81 ms, Matrix = 152 × 154, Slice Thickness = 4 mm, Field of View (FOV) = 230 × 230 mm^2^, Total Scan Time = 2 min and 2 s.
  Additionally, the following sequences were also used: 3D T1-weighted (T1w) Turbo Field Echo (TFE), 3D T2 FLAIR (Fluid-Attenuated Inversion Recovery) weighted Turbo Field Echo (TFE), Susceptibility-Weighted Imaging (SWI), Dynamic Susceptibility Perfusion Imaging (DSC Perfusion), Multivoxel MR Spectroscopy (MRS), Post-contrast 3D T1-weighted (T1w) Turbo Field Echo (TFE)

### Analysis

The study involved analyzing imaging data in Digital Imaging and Communications in Medicine (DICOM) format, which included APT-weighted (APTw) and other sequences. The data was processed using a workstation, and a symmetrical alignment was applied to ensure consistency.

Two experienced neuroradiologists, with 9 and 35 years of experience respectively, performed manual segmentation of the neoplasms. They delineated the tumor boundaries on each image slice in all sequences, identifying the 2D regions of interest (ROIs) corresponding to the tumors. This meticulous segmentation was critical for precise quantitative analysis. The neuroradiologists were blinded to the pathological diagnosis of the tumors to prevent bias during imaging analysis.

Following segmentation, imaging features of the tumors were comprehensively analyzed. This analysis encompassed tumor location, size, shape, enhancement pattern, necrosis, and edema. Specifically, APTw images were scrutinized for a high APT signal, indicative of mobile proteins and peptides within the tumor tissue.

For the APT quantification, after water frequency shift correction, magnetization transfer component and the APT (Δω = 3.5 ppm) component, asymmetrical MT ratio (MTRasym) analysis was performed. Arterial Spin Labeling (ASL) MRI was used to measure relative cerebral blood flow (rCBF) in the tumor tissue region compared to a reference region in the normal-appearing contralateral brain. rCBF values less than 1 were indicative of low ASL perfusion.

The primary objective was to distinguish pilocytic tumors from non-PA posterior fossa tumors. To achieve this, a two-sided Fisher's exact test was performed to calculate the p-value, given the binary nature of the APT-ASL mismatch data (yes/no). Fisher's exact test was appropriate for the small sample sizes or when data cells had expected frequencies less than 5. An ROC (Receiver Operating Characteristic) curve was generated to assess the diagnostic value of the ASL-APT mismatch for differentiating tumors. Apparent Diffusion Coefficient (ADC) values from diffusion images of the tumor were analyzed to assess tissue characteristics and aid in differentiation. Two-sample t-test is used to compare the ADC (Apparent Diffusion Coefficient) values between pilocytic tumors and non-pilocytic tumors.

## Results

**Figure 2:**
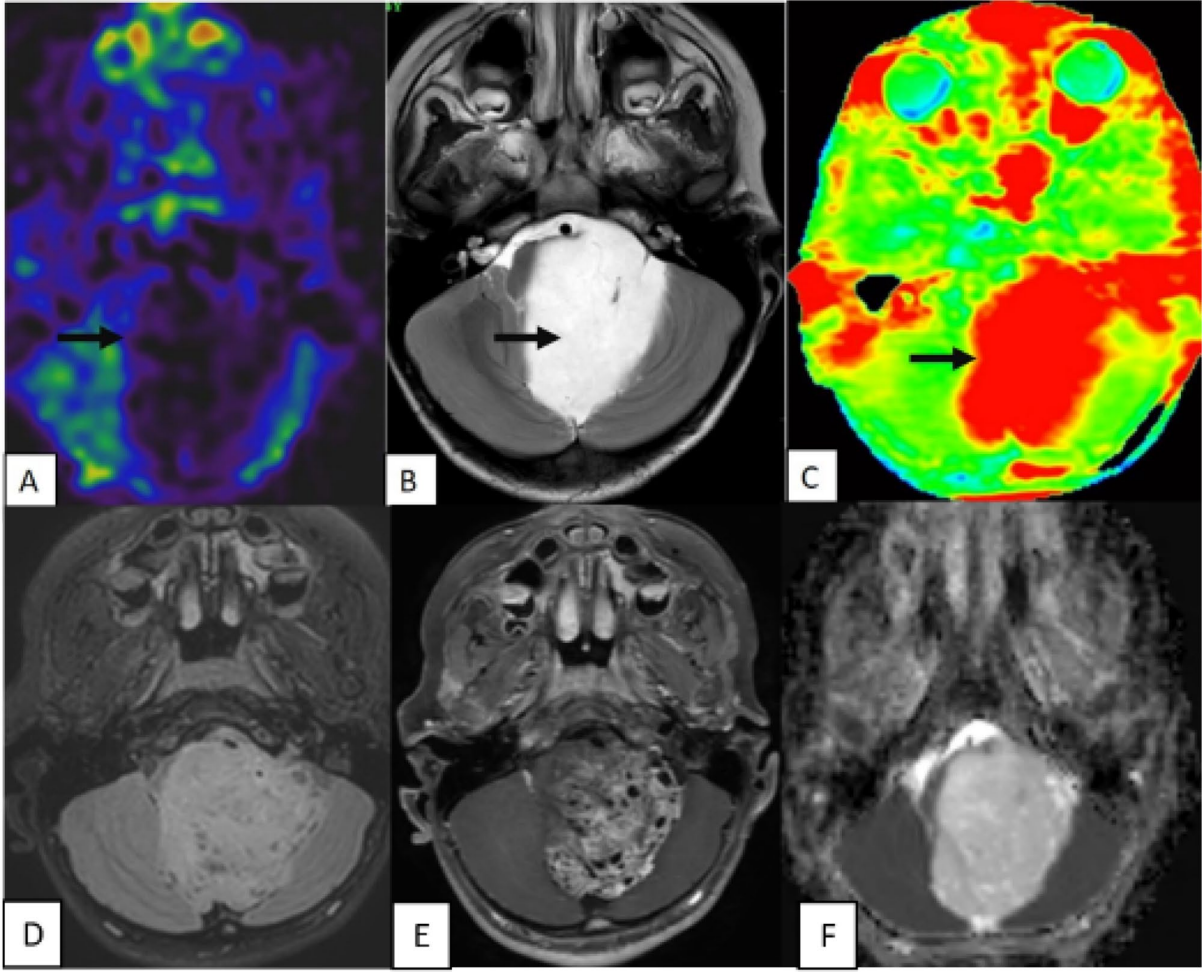
A well-defined T2 hyperintense (**B**) large solid non cystic mass lesion seen involving the left side pons, left cerebellar hemisphere. Lesion showed diffuse hypoperfusion in ASL (**A**) and diffuse high values in Amide Proton Transfer Imaging (**C**). Amide Proton Transfer-Arterial spinal labeling (APT-ASL) mismatch seen. Lesion appears T2 FLAIR (**D**) hyperintense, and shows aggressive heterogenous enhancement (**E**), with no significant diffusion restriction (F, high apparent diffusion coefficient-ADC). Conventional imaging differential diagnosis were ependymoma, pilocytic astrocytoma. This is a histologically proven Grade 1 Pilocytic Astrocytoma.

**Figure 3:**
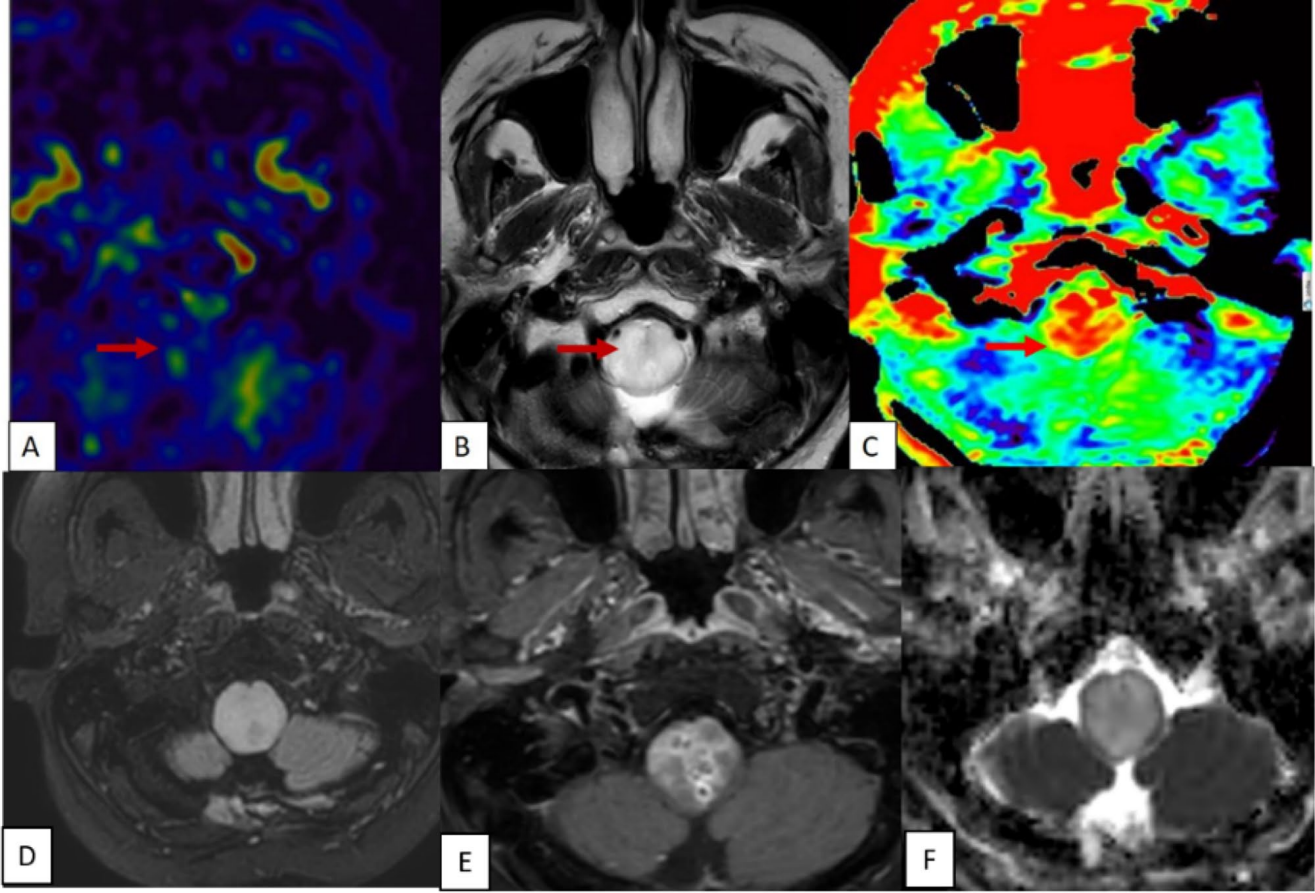
A T2 hyperintense (**B**) solid expansile mass lesion seen involving the medulla. Lesion showed diffuse hypoperfusion in ASL (**A**) and diffuse high values (**C**) in Amide Proton Transfer Imaging. Amide Proton Transfer-Arterial spinal labeling (APT-ASL) mismatch seen. Lesion appears T2 FLAIR (**D**) hyperintense, and shows heterogenous enhancement (**E**), with no significant diffusion restriction (**F**). Imaging differentials based upon on conventional sequences were astrocytoma, ependymoma. But the final histological diagnosis was Grade 1 Pilocytic Astrocytoma.

**Figure 4:**
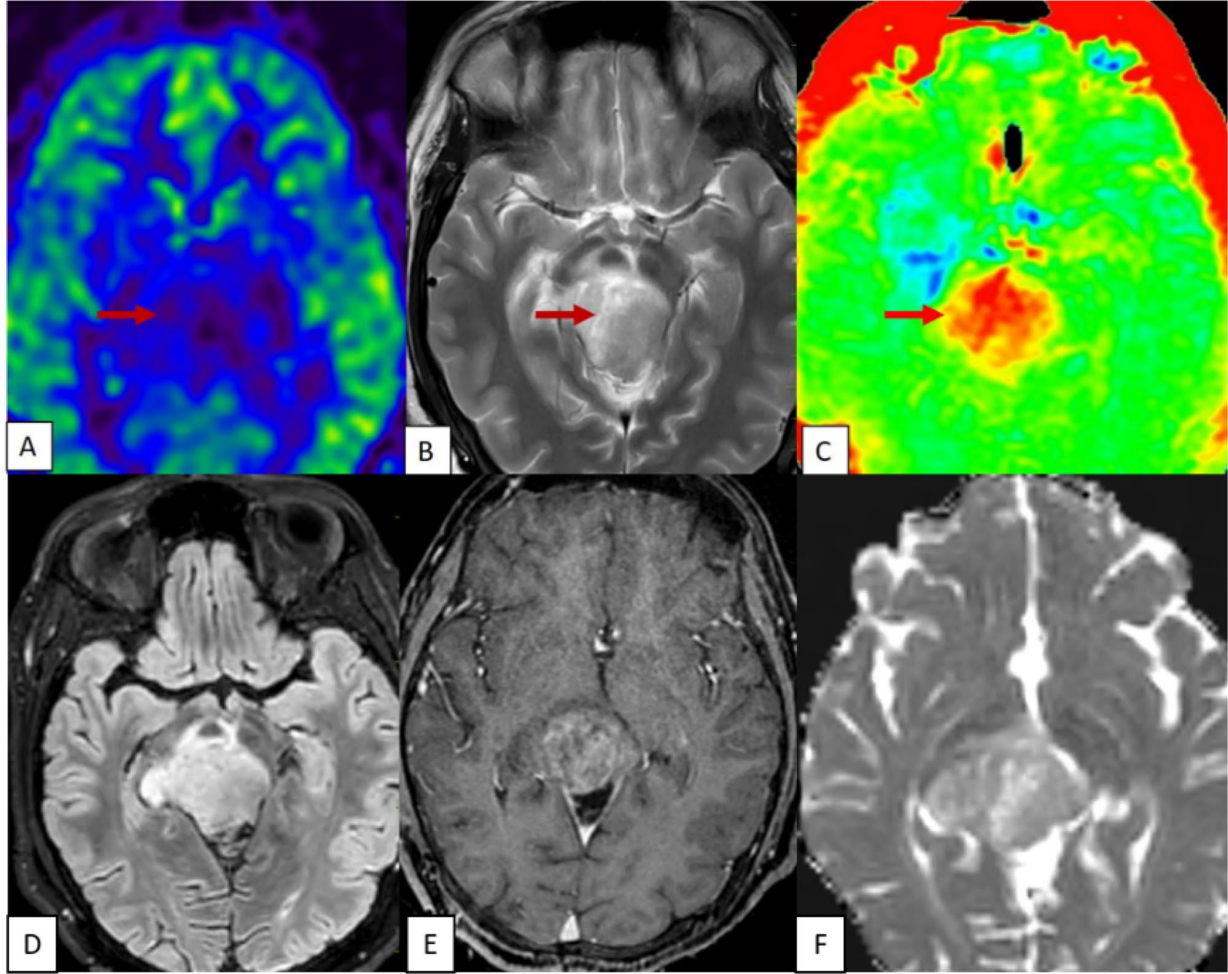
A T2 hyperintense solid expansile mass lesion (**B**) seen involving the midbrain. Lesion showed diffuse hypoperfusion in ASL (**A**) and diffuse high values (**C**) in Amide Proton Transfer Imaging. Conventional imaging diagnosis was diffuse midline glioma, high grade glioma. Lesion appears T2 FLAIR (**D**) hyperintense, and shows heterogenous enhancement (**E**), with patchy diffusion restriction (**F**). But the histological diagnosis was Grade 1 Pilocytic Astrocytoma. Amide Proton Transfer-Arterial spinal labeling (APT-ASL) mismatch seen.

**Figure 5:**
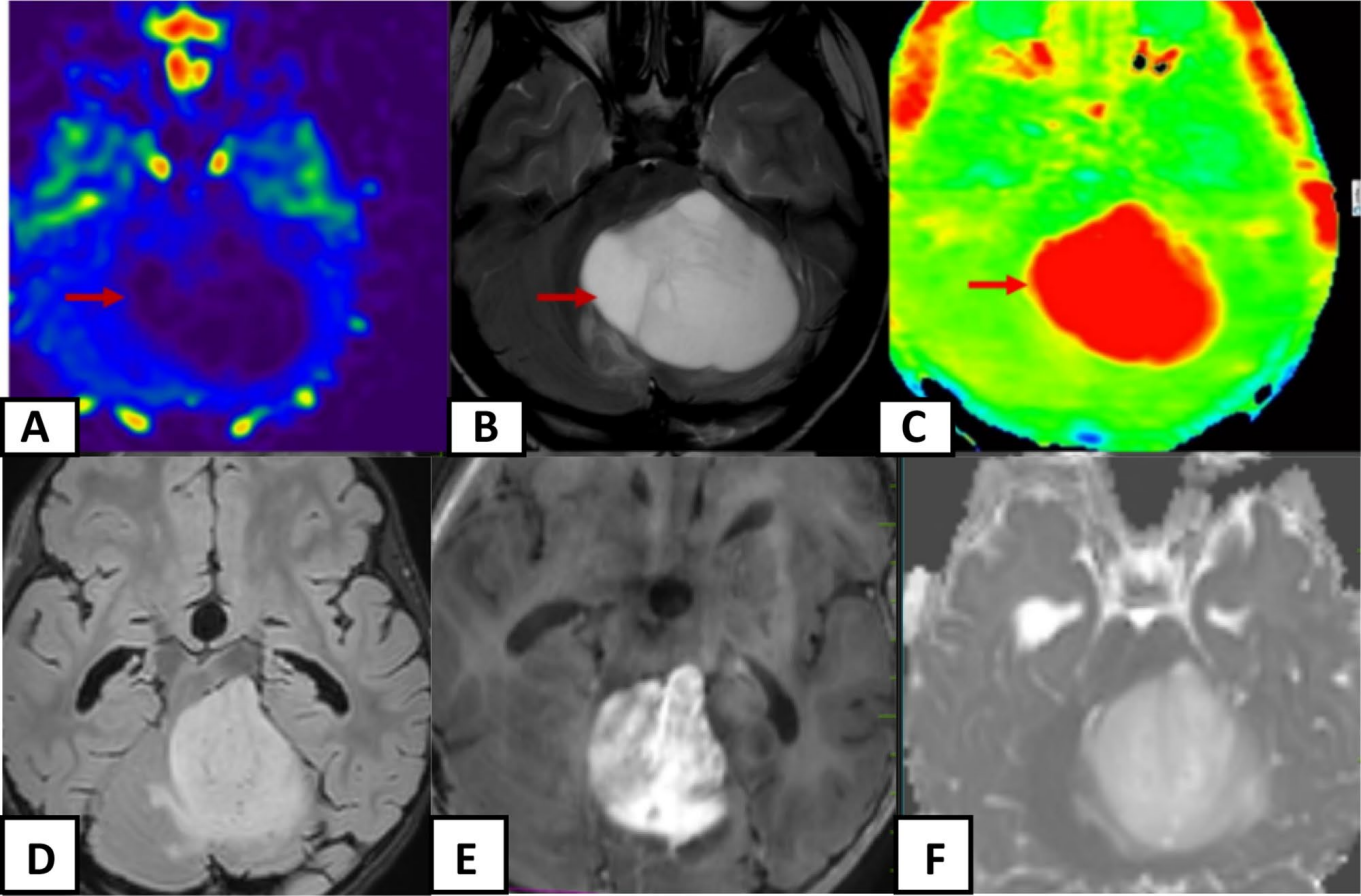
A T2 hyperintense (**B**) predominantly solid mass lesion seen involving the cerebellum, left middle cerebellar peduncle Lesion showed diffuse hypoperfusion in ASL (**A**) and diffuse high values in Amide Proton Transfer Imaging (**C**). Lesion showed intense enhancement (**E**), with no significant diffusion restriction (**F**). This is a histologically proven Grade 1 Pilocytic Astrocytoma. Amide Proton Transfer -Arterial spinal labeling (APT-ASL) mismatch seen.

**Figure 6:**
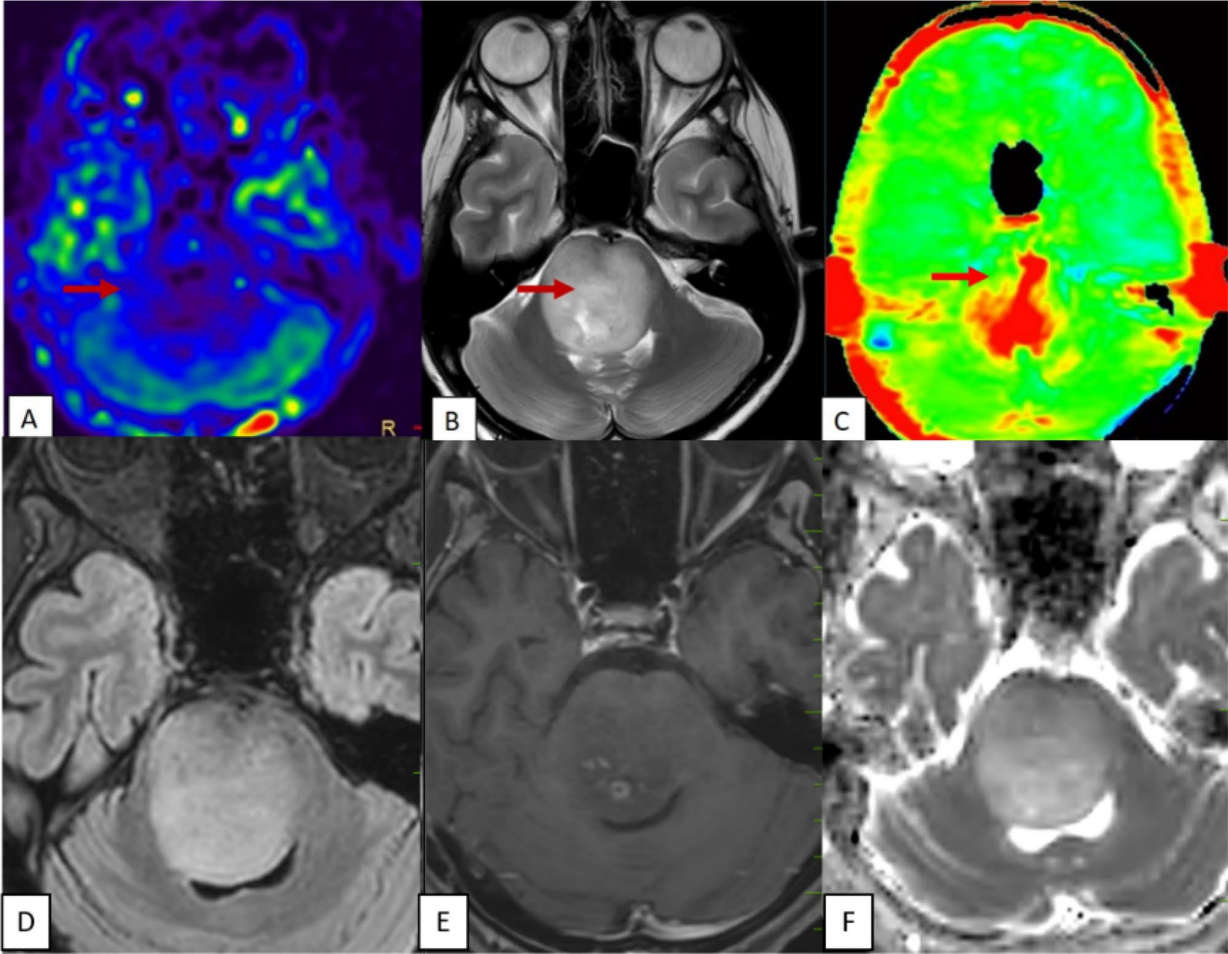
A T2 hyperintense (B) solid expansile mass lesion seen involving the Pons. Lesion showed diffuse hypoperfusion in ASL (**A**) and diffuse high values in Amide Proton Transfer Imaging. (**C**) Lesion is T2 FLAIR hyperintense, (**D**) with minimal enhancement (**E**) and no significant diffusion restriction (**F**). This case was mistaken as diffuse midline glioma on conventional T2 sequences based upon location and pons expansion, but it was later histologically proved as Grade 1 Pilocytic Astrocytoma. Amide Proton Transfer -Arterial spinal labeling (APT-ASL) mismatch seen.

The study’s results, presented in Table 1, revealed that all 25 cases of Pilocytic Astrocytoma (PA) exhibited a consistent and diffuse increase in APT signal intensity (APTSI) values in the Amide Proton Transfer imaging (APTw). These APTSI values reached a threshold of greater than or equal to 4. Moreover, in arterial spin labeling (ASL) perfusion imaging (Figs. 2, 3, 4, 5 and 6), all PA lesions showed low cerebral blood flow (CBF) values. This consistent pattern indicated a clear mismatch between APTSI and ASL CBF values, observed in every case of Pilocytic Astrocytoma.

**Table 1:** Results of the study.

	Pilocytic tumours	Non Pilocytic tumors
Number of cases	25	25 [diffuse midline glioma-5, Ependymoma-4, Medulloblastoma-4, Diffuse astrocytoma (grade 2 and 3)-7, Atypical teratoid rhabdoid tumor-1, Embryonal tumor with multi-layered rossetes-1, Pleomorphic xanthoastrocytoma-3]
APT-ASL mismatch	25 (100%)	0 (0%)
ADC (Mean)	1.58 × 10^–3^ mm^2^/s	1.09 × 10^–3^ mm^2^/s
Age (Mean, Range)	13.2 (2 to 40 years)	16.6 (2 to 63 years)
Sex	Males—10	Males—13
	Females—15	Females—12
Location of tumors	Brainstem—13	Brainstem—8
	Cerebellum—4	Cerebellum—8
	Cerebral hemsipheres—4	Cerebral hemispheres—8
	Optic chiasm—1	Thalamus—1
	Thalamus—3

**Figure 7:**
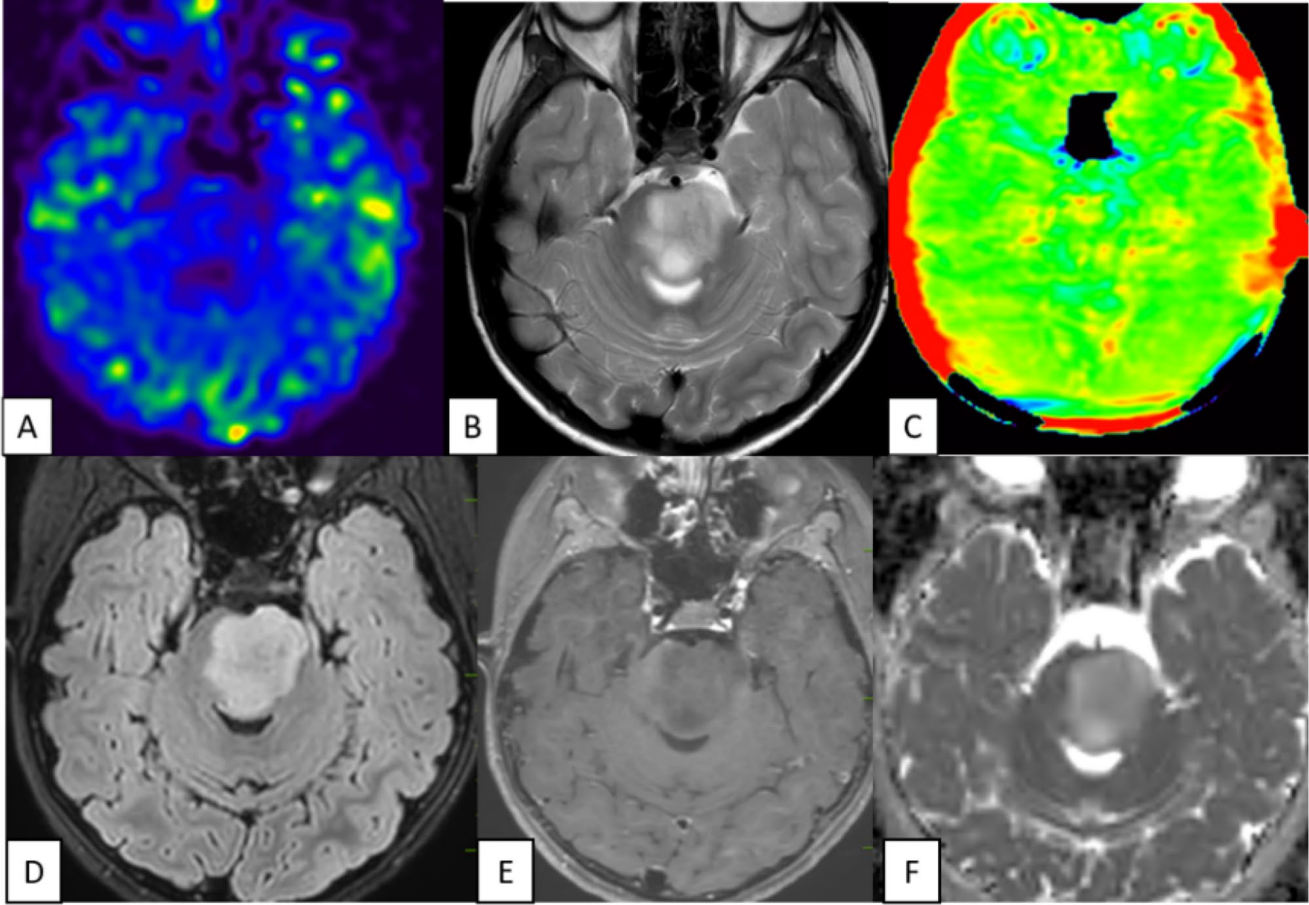
T2 hyperintense (**A**) lobulated mass lesion noted at the pons. ASL showed complete hypoperfusion (**B**). Amide proton transfer also showed diffuse low values (**C**). No significant mismatch seen between ASL and amide proton transfer in this case. Conventional imaging was mimicking as pilocytic astrocytoma due its circumscribed homogenous T2 hyperintense signal appearance. Lesion is T2 FLAIR hyperintense, (**D**) with no enhancement (**E**) and is showing patchy diffusion restriction (**F**). This case was histopathologically proven as diffuse midline glioma H3 K27 altered.

**Figure 8:**
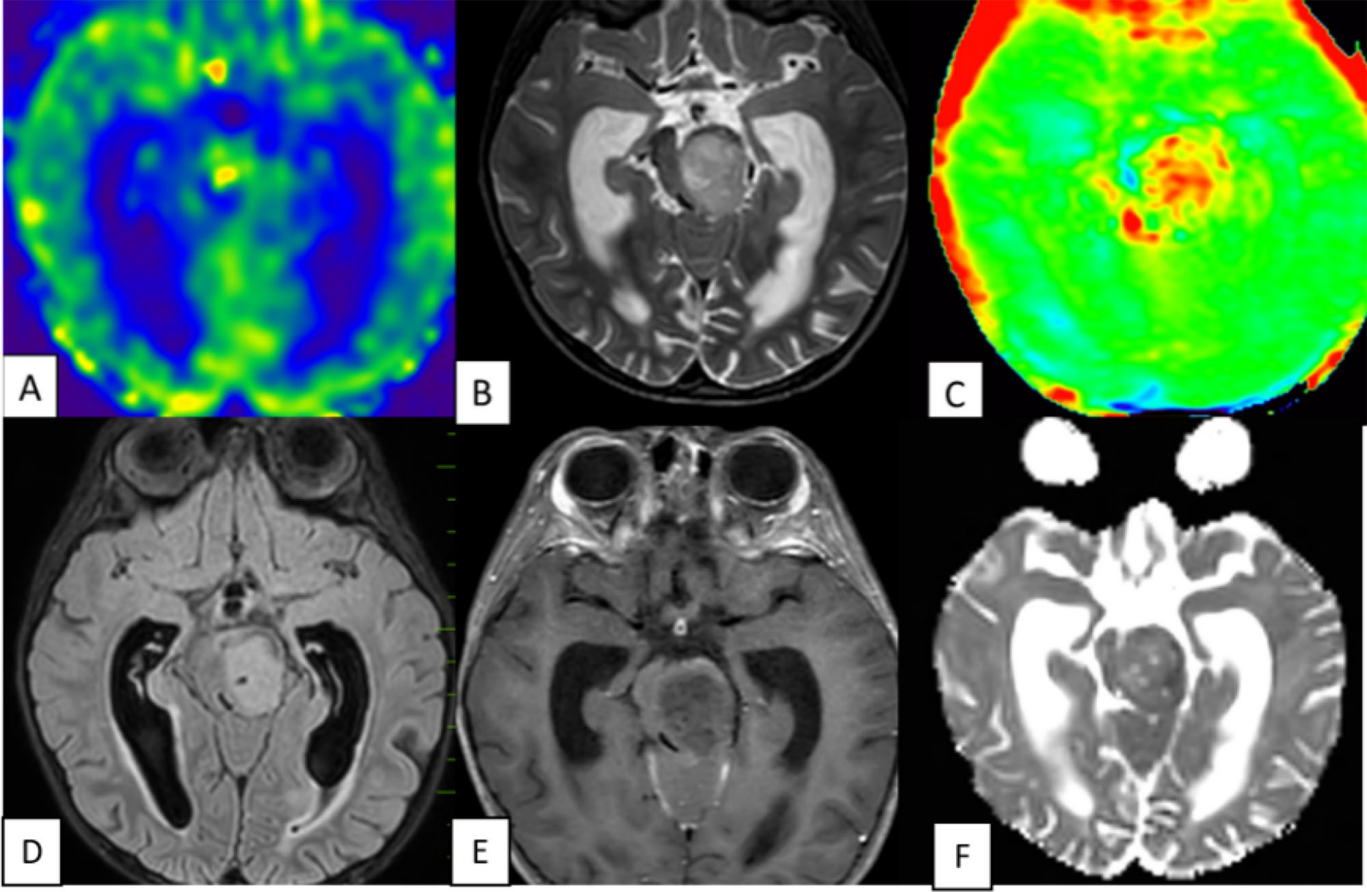
T2 MRI (**B**) also showed elevated values hyperintense solid mass lesion noted at the left side midbrain. ASL (**A**) showed raised perfusion at the lesion. Amide proton transfer (**C**) showed elevated values corresponding to the regions, with raised perfusion in ASL. No significant mismatch seen between ASL and amide proton transfer. Lesion is T2 FLAIR hyperintense, (**D**) with no enhancement (**E**) and patchy diffusion restriction (**F**). This case was misinterpreted as pilocytic astrocytoma in conventional imaging, due its circumscribed homogenous T2 hyperintense signal lobulated appearance. This was diagnosed histopathologically as embryonal tumor with multilayered rosettes (ETMR).

On the contrary, the remaining 25 patients with non-pilocytic tumors, did not display such a mismatch between APTSI and ASL CBF values (Figs. 7, 8). These findings suggest that the APT-ASL mismatch is specific to Pilocytic Astrocytoma, making it a valuable diagnostic marker to distinguish it from other brain tumors.

The APT-ASL mismatch was observed in all 25 cases of pilocytic tumors and none of the non-pilocytic tumors. The statistical analysis using a two-sided Fisher's exact test yielded an extremely low p-value, less than 0.0001, indicating a perfect association between the APT-ASL mismatch and pilocytic tumors in the dataset. This statistically significant association suggests that the APT-ASL mismatch could serve as a reliable diagnostic marker for distinguishing pilocytic astrocytomas from other brain tumors.

As the observed association resulted in no false positives or false negatives, the sensitivity and specificity values were both 100% for all cutoff points. Consequently, the ROC curve became a perfect vertical line, passing through the points (0,1) and (1,1) on the sensitivity–specificity plane. The mean ADC value for pilocytic tumors was 1.58 with a standard deviation of 0.371, while for non-pilocytic tumors, the mean ADC value was 1.08 with a standard deviation of 0.50. The calculated t-value of approximately − 4.50 with 42 degrees of freedom yielded a p-value of approximately 0.0000132. The obtained p-value indicates strong evidence against the null hypothesis, suggesting a significant difference in ADC values between pilocytic and non-pilocytic tumors.

## Discussion

Amide Proton Transfer (APT) imaging is a relatively new technique that uses Magnetic Resonance Imaging to measure the chemical exchange between water protons and the amide protons of mobile proteins and peptides in tissues^6^. APT imaging can provide information about the concentration and chemical exchange rate of amide protons in tissues, which can help to identify tissue characteristics such as protein content and pH levels.

APT imaging has been studied in various medical applications, including brain tumor imaging. In gliomas, the most common primary brain tumors, APT imaging has been shown to be a useful tool for differentiating between low-grade and high-grade tumors. High-grade gliomas tend to have higher APT signal intensity due to their increased protein content, while low-grade gliomas tend to have lower APT signal intensity due to their lower protein content^7^. Recent retrospective studies conducted by Jiang et al. have demonstrated the potential of APTw imaging as an imaging biomarker for identifying important genetic characteristics of gliomas. In particular, they found that the APTw signal can help distinguish between IDH mutation status in low-grade gliomas^8,10^ and MGMT methylation status in high-grade gliomas^9–11^. The results indicated that IDH-wildtype lesions typically exhibited higher APTw intensities compared to IDH-mutant lesions, which is consistent with laboratory research showing downregulation of protein expression in mutant IDH1-driven glioma cells. These initial findings, along with subsequent confirmatory results, hold great promise. If further validated through rigorous clinical studies, APTw imaging could potentially enable rapid and non-invasive determination of genetic biomarkers before surgery, aiding in more precise treatment planning for glioma patients.

The use of APT imaging in brain tumor imaging has several potential benefits. Unlike conventional MRI techniques, APT imaging does not require the use of a contrast agent, which can have potential side effects^12^. Additionally, APT imaging has been shown to be more sensitive than conventional MRI techniques in detecting tumor tissue, particularly in regions where conventional MRI can be limited by artifacts or other imaging challenges^13^.

In this study we have investigated the use of APT imaging in the diagnosis of Pilocytic Astrocytomas, a type of low-grade brain tumor that can be difficult to differentiate from other tumor types based on conventional MRI. We found that APT imaging could provide a unique imaging appearance in Pilocytic Astrocytomas, characterized by high APTSI values and low signal in ASL perfusion imaging. This mismatch between APT and ASL perfusion imaging could be a biomarker for Pilocytic Astrocytomas and could aid in their diagnosis and differentiation from other tumor types.

We have noted that the high APT values in Pilocytic Astrocytomas may be due to their high intracellular and extracellular matrix protein content, as well as microcystic changes. The low ASL perfusion signal may be due to the low-grade nature of Pilocytic Astrocytomas. We suggested that the unique imaging appearance of Pilocytic Astrocytomas on APT and ASL perfusion imaging could be a useful diagnostic tool, particularly in cases where conventional MRI is inconclusive.

While the study showed promising results regarding the use of APT imaging in the diagnosis of Pilocytic Astrocytomas, it is important to acknowledge some limitations of the study. The study was a retrospective analysis of a small number of patients from a single institution, which may limit the generalizability of the findings to larger populations or other settings. APT imaging is a relatively new technique, and its reproducibility and reliability across different MRI machines and settings are not well established. This may affect the consistency and accuracy of the APT imaging measurements and their interpretation. Finally, while APT imaging does not require the use of a contrast agent, it still requires special MRI sequences and additional post-processing, which may increase the scanning time and cost, as well as the technical expertise needed to perform and interpret the scans. Therefore, while the results of the study are promising, further research with larger and more diverse populations, including comparisons with other imaging modalities and histopathology, as well as standardization of APT imaging protocols, is needed to fully validate the diagnostic utility of APT imaging in the diagnosis and differentiation of pilocystic astrocytoma. Hence, external validation of the study findings with larger and independent patient cohorts will strengthen the credibility and reliability of APT imaging as a diagnostic biomarker.

In addition, the results of the analysis using the two-sample t-test on ADC values from pilocytic and non-pilocytic brain tumors revealed a statistically significant difference between the two groups (p < 0.05). This finding suggests that there are distinct ADC characteristics associated with different types of brain tumors. These results are consistent with previous research^14,15^ indicating that ADC values can be useful in differentiating various tumor types based on their cellular composition and microstructure. The ability to non-invasively differentiate between pilocytic and non-pilocytic tumors using ADC values could have clinical implications, potentially aiding in accurate diagnosis and treatment planning.

In conclusion, Amide Proton Transfer imaging is a promising technique for the diagnosis and differentiation of brain tumors, including Pilocytic Astrocytomas. The unique imaging appearance of Pilocytic Astrocytomas on APT and ASL perfusion imaging could be a useful diagnostic tool, particularly in cases where conventional MRI is inconclusive. Further research is needed to validate the use of APT imaging in clinical practice, but its potential benefits, including its non-invasiveness and lack of contrast agent use, make it an exciting area of research in the field of medical imaging.

## Conclusion

Our study demonstrates the potential of APTw and ASL imaging as valuable tools for differentiating pilocytic astrocytomas from other brain tumors. The high proton transfer values observed in pilocytic astrocytomas in this study are likely due to the high protein content within the tumor cells and extracellular matrix, as well as microcystic changes. The low CBF in ASL is consistent with the low-grade nature of pilocytic astrocytomas. The mismatch between APT signal intensity and ASL CBF seen in all cases of pilocytic astrocytomas in our study highlights the unique imaging appearance of these tumors, and may serve as a biomarker for diagnosis.

## Data Availability

Datasets generated and/or analyzed during this study are available from the corresponding author upon reasonable request.
